# EHMTI-0376. Trigeminovascular sensitisation by chronic subdural haemorrhage: four clinical paediatric cases

**DOI:** 10.1186/1129-2377-15-S1-K6

**Published:** 2014-09-18

**Authors:** W Squier, J Mack

**Affiliations:** 1 Neuropathology, University of Oxford, Oxford, UK; 2 Radiology, Penn State Hershey Medical Center, Hershey Pennsylvania, USA

## Background

Sensitisation of the trigeminal nerve (TG) has been extensively studied as a potential mechanism of migraine. Meningeal nociceptors project onto second order neurones of the trigeminocervical complex (TCC) via glutamate neurotransmission. Sensitisation of trigeminal afferents causes massive release of glutamate and central sensitisation. The TCC connects with the VII, IX and Xth cranial nerve nuclei, and participates in the trigeminocardiac reflex which is particularly important in the young .

## Aim

In the laboratory, application of 'inflammatory soup' causes increased trigeminal sensitisation and increased responsiveness so that dural afferents can be strongly activated by mechanical and other stimuli that initially had evoked little or no response. We wished to show that bleeding in the human dura, which produces an inflammatory response and increased mast cell numbers, may sensitise the trigeminal system.

## Method

We examined our autopsy database for infants and children with chronic subdural haemorrhage who collapsed and died unexpectedly.(Local Research Ethics and NHS research governance approved the study (10/H0604/83) (Ref 6321)).

## Results

We identified four patients with chronic dural haemorrhage who died following following TG stimulation; one by hypertension and three by oronasal stimulation during choking. All had selective necrosis in the spinal nucleus of the TCC at autopsy.

## Conclusion

We suggest these cases represent excitotoxic damage in the presence of sensitisation by chronic dural haemorrhage. Collapse may have been mediated by the trigeminocardiac reflex resulting in bradycardia, hypotension and apnoea. Exaggeration of this protective response has been implicated in Sudden Infant Death Syndrome.

No conflict of interest.

